# Chemical contamination and nutritional degradation of freshwater fish marketed in West Kazakhstan

**DOI:** 10.14202/vetworld.2026.1533-1549

**Published:** 2026-04-24

**Authors:** Askhat Zhumabayev, Birzhan Nurgaliyev, Ilana Abirova, Zhenis Kushmukhanov, Zhangeldi Ussenov, Aigerim Kozhayeva, Abzal Kenesovich Kereyev, Akatova Rysbike, Nurgaliyeva Mira

**Affiliations:** 1 Institute of Veterinary and Agrotechnology, West Kazakhstan Agrarian and Technical University named after Zhangir Khan, Uralsk 090009, Republic of Kazakhstan; 2 Shakarim Lab, Shakarim University, Semey 071412, Republic of Kazakhstan

**Keywords:** biochemical degradation, cadmium contamination, food safety, freshwater fish, heavy metal toxicity, nutritional quality, public health risk, West Kazakhstan

## Abstract

**Background and Aim:**

Freshwater fish are an important source of high-quality protein, essential amino acids, omega-3 polyunsaturated fatty acids, vitamins, and minerals. However, contamination of aquatic ecosystems with toxic elements and radionuclides may compromise both food safety and the nutritional and biochemical integrity of fish. This study aimed to evaluate the association between toxic elements, radionuclides, and biochemical degradation of freshwater fish marketed for human consumption in West Kazakhstan.

**Materials and Methods:**

A cross-sectional observational study was conducted from April to September 2025. A total of 800 samples were analyzed, including 500 fresh freshwater fish specimens representing five species and 300 processed fish products collected from retail outlets supplied by 15 water bodies and one aquaculture facility. Concentrations of lead (Pb), cadmium (Cd), arsenic (As), and mercury (Hg) were determined using validated standard methods. Radionuclide activity of cesium-137 (Cs-137) and strontium-90 (Sr-90) was also measured. For biochemical analysis, fish were divided into a control group (no detectable contamination; n = 50) and a contaminated group (low-dose Pb and/or Cd detected; n = 50). Nutritional composition, mineral profile, vitamin content, amino acid composition, and fatty acid profiles were analyzed using standardized laboratory techniques. Statistical analysis included effect size estimation, false discovery rate correction, and multivariate modeling.

**Results:**

Pb and Cd were detected in fish from 7 of 15 water bodies, with localized exceedances reaching 1.15 mg/kg and 0.245 mg/kg, respectively. As and Hg were not detected, while radionuclide activity remained within permissible limits. Compared with the control group, contaminated fish showed significant reductions in lipid content (−26.3%) and energy value (−12.7%), along with decreases in essential minerals, vitamins, and total amino acid content (−15.8%) (p ≤ 0.05). Omega-3 polyunsaturated fatty acids, particularly eicosapentaenoic acid and docosahexaenoic acid, were markedly reduced. Multivariate analysis confirmed that contamination status was an independent predictor of biochemical deterioration irrespective of species.

**Conclusion:**

The findings demonstrate that compliance with regulatory contaminant limits does not guarantee preservation of nutritional quality. Chronic low-dose exposure to Pb and Cd is associated with substantial biochemical degradation of fish muscle tissue, even when organoleptic quality remains acceptable. These results highlight an overlooked dimension of food safety and support the integration of biochemical and nutritional indicators into routine monitoring frameworks to improve consumer protection.

## INTRODUCTION

Fish and fish products are essential components of the human diet due to their high content of high-quality protein, essential amino acids, long-chain omega-3 polyunsaturated fatty acids (PUFAs), vitamins, and minerals. Regular fish consumption is associated with substantial health benefits; however, the nutritional value of fish may be compromised by contamination of aquatic ecosystems, which can alter biochemical composition and reduce the overall dietary quality of aquatic food resources [1, 2].

Among environmental contaminants, toxic elements such as lead (Pb) and cadmium (Cd), as well as radionuclides, are of particular concern due to their persistence, bioaccumulation potential, and adverse effects on biological systems and human health. Recent studies have demonstrated that Pb and Cd concentrations in freshwater fish may approach or exceed regulatory limits, particularly in regions characterized by intensive industrial and agricultural activities, thereby raising concerns regarding both food safety and long-term dietary exposure [3, 4]. In Kazakhstan and Central Asia, despite increasing environmental pressure associated with industrial development, agricultural intensification, and transboundary water systems, comprehensive regional assessments of fish safety and quality remain limited. In particular, integrative studies simultaneously addressing contaminant burden and contamination-induced alterations in the nutritional and biochemical quality of freshwater fish marketed for human consumption are still scarce [5].

Most fish safety studies and monitoring programs primarily focus on contaminant concentrations and compliance with regulatory limits. While such approaches are essential for food safety control, they provide limited insight into contamination-induced changes in the nutritional and biological quality of fish. Increasing evidence indicates that chronic exposure to toxic elements can alter metabolic processes in fish, leading to qualitative biochemical degradation that is not detected by conventional monitoring indicators [6].

Biochemical components such as amino acids, fatty acids, antioxidant vitamins, and essential minerals are sensitive indicators of metabolic integrity and nutritional value. These parameters are directly involved in protein synthesis, membrane structure, oxidative balance, and energy metabolism and may respond to contaminant exposure even when gross composition and organoleptic quality remain acceptable [7, 8]. However, studies that simultaneously integrate toxic elements, radionuclides, and detailed biochemical characterization of fish muscle tissue within a single analytical framework remain scarce, particularly for freshwater fish marketed for human consumption in Central Asia.

Unlike conventional surveillance studies that focus primarily on compliance with maximum permissible limits, the present study integrates toxicological findings with a comprehensive biochemical assessment of fish muscle tissue, enabling evaluation of contamination-induced deterioration in nutritional and biological value. The simultaneous evaluation of protein, lipid, mineral, vitamin, amino acid, and fatty acid profiles provides an integrated biochemical signature of contamination-related metabolic disruption, which has rarely been reported for freshwater fish in Central Asia.

The inclusion of radionuclide assessment alongside heavy metals extends the scope of food safety evaluation beyond conventional chemical contaminants and contributes to a more holistic assessment of environmental stressors affecting fish quality. This study provides one of the first region-specific datasets linking chemical contamination to nutritional degradation of commercially important freshwater fish in West Kazakhstan.

Despite extensive global research on heavy metal contamination in fish, current evidence remains largely focused on the quantification of contaminant concentrations and their compliance with regulatory thresholds. Such approaches, while essential for food safety monitoring, provide limited insight into contamination-induced alterations in the nutritional and biochemical integrity of fish. In particular, most studies evaluate proximate composition parameters (e.g., protein, lipid, moisture) without examining deeper biochemical indicators such as amino acid profiles, fatty acid composition, vitamin status, and mineral balance, which are more sensitive to metabolic disruption. Furthermore, the majority of existing investigations consider either chemical contaminants or nutritional attributes in isolation, rather than integrating both dimensions within a unified analytical framework.

In the context of Central Asia, and specifically West Kazakhstan, there is a marked scarcity of region-specific, comprehensive datasets that simultaneously assess toxic elements, radionuclides, and detailed biochemical composition of freshwater fish marketed for human consumption. Existing studies from the region are fragmented, often limited in scope, and rarely address the relationship between environmental contamination and functional nutritional quality. Additionally, the potential for sub-threshold contamination to induce significant biochemical deterioration remains insufficiently explored, despite increasing evidence that chronic low-dose exposure may alter metabolic processes without exceeding regulatory limits. This gap is further compounded by the lack of multivariate analytical approaches capable of distinguishing contamination effects from species-specific variability. Consequently, a critical knowledge gap exists in understanding how environmental contaminants influence the biological and nutritional value of fish beyond conventional safety indicators.

Accordingly, the present study was designed to address these limitations by integrating toxicological, radiological, and comprehensive biochemical analyses within a single analytical framework. The primary aim was to evaluate the association between toxic elements, radionuclides, and contamination-induced biochemical degradation of freshwater fish muscle tissue marketed for human consumption in West Kazakhstan. Specifically, the study sought to (i) determine the concentrations and spatial distribution of lead, cadmium, arsenic, mercury, cesium-137, and strontium-90 in commercially available fish; (ii) assess the impact of contaminant exposure on key nutritional and biochemical parameters, including protein, lipid content, energy value, mineral composition, vitamin levels, amino acid profiles, and fatty acid composition; and (iii) examine whether contamination status acts as an independent predictor of biochemical deterioration irrespective of species differences.

In addition, the study aimed to move beyond conventional compliance-based food safety assessment by evaluating whether sub-threshold contamination is associated with measurable reductions in nutritional quality. By combining quantitative contaminant analysis with detailed biochemical profiling and multivariate statistical modeling, this research provides an integrative assessment of fish quality that reflects both toxicological safety and functional nutritional value. The findings are intended to support the development of more comprehensive monitoring strategies that incorporate biochemical indicators alongside traditional contaminant thresholds, thereby improving risk assessment and consumer protection within a One Health framework.

## MATERIALS AND METHODS

### Ethical approval

Ethical approval was not required for this study because it used only commercially available freshwater fish and processed fish products purchased from retail outlets in Uralsk, West Kazakhstan, and did not involve any live-animal experimentation, animal handling, capture, invasive sampling, or procedures causing pain, distress, or behavioral manipulation. The investigation was conducted as a market-based observational food quality and safety assessment under real consumer exposure conditions. Samples were obtained from retail outlets supplied by regional freshwater bodies and one aquaculture facility, and all laboratory analyses were performed on post-harvest edible muscle tissue after purchase. Therefore, the study did not fall within the scope of institutional animal ethics review requirements for experimental animal research. Nevertheless, all procedures were carried out in accordance with good laboratory practice and relevant national standards for sample handling, transport, storage, and analytical testing to ensure scientific integrity and biosafety.

### Study period and location

Sampling was carried out from April to September 2025 in the city of Uralsk (West Kazakhstan) at the Institute of Veterinary Science and Agrotechnology, Zhangir Khan West Kazakhstan Agrarian Technical University. Fish and fish products were obtained from retail outlets (“Ayazhan”, “Mirlan”, and “El-Yrysy”), which were supplied by freshwater water bodies located within the West Kazakhstan region.

The study area is characterized by mixed anthropogenic pressures, including urban activities, agricultural practices, and localized industrial sources, all of which may contribute to chemical contamination of aquatic ecosystems.

The extended sampling period was selected to minimize short-term variability and to obtain representative data across the main fishing and marketing season, thereby reflecting real-market exposure conditions. Retail batches were randomly selected from available deliveries to minimize selection bias and improve representativeness.

### Study design

This study was designed as a cross-sectional observational investigation to evaluate the association between chemical contamination (toxic elements and radionuclides) and the biochemical composition of fish muscle tissue.

The objective of the study was not to establish causal relationships, but rather to identify contamination-associated biochemical alterations under real-market conditions that reflect fish and fish products available to consumers. Accordingly, the study design was focused on exposure–response relationships rather than mechanistic causality.

### Fish species selection

Several commercially important freshwater fish species were selected based on their nutritional relevance, trophic diversity, and widespread consumption in the region, including common carp (*Cyprinus carpio*), asp (*Aspius aspius*), crucian carp (*Carassius carassius*), pike perch (*Sander lucioperca*), bream (*Abramis brama*), and sabrefish (*Pelecus cultratus*). Sabrefish was analyzed only among processed retail products.

Determination of heavy metals and radionuclides was conducted primarily using fresh fish collected from natural freshwater bodies and one aquaculture facility. Processed fish products purchased from retail outlets were analyzed exclusively for consumer safety assessment and were not included in evaluations of environmental contamination.

The selected species represent different feeding strategies and trophic levels, allowing the assessment of potential interspecific differences in contaminant accumulation and associated biochemical responses.

Species identity was retained in the dataset to permit supplementary interspecific comparisons and exploratory multivariate modeling.

### Sample size determination

A total of 800 fish samples were analyzed in this study. Of these, 500 samples consisted of fresh freshwater fish representing five commercially important species: common carp (*C. carpio*; n = 100), asp (*A. aspius*; n = 100), crucian carp (*C. carassius*; n = 100), pike perch (*S. lucioperca*; n = 100), and bream (*A. brama*; n = 100). All fresh fish samples were analyzed for the presence of toxic elements and radionuclides.

In addition, 300 samples of processed fish products were collected, including dried bream (n = 100), dried sabrefish (*P. cultratus*; n = 100), and smoked bream (n = 100). All samples were obtained from three retail outlets (“Ayazhan”, “Mirlan”, and “El-Yrysy”), which were supplied by 15 freshwater water bodies in West Kazakhstan (Derköl, Solyanka, Embulatovka, Shezhin-1, Shezhin-2, Barbastau, Muratsai, Berezovka, Shagan, Bagyrlay, Ashysai, Shyngyrlau, Shiderty, Kaldygayty, and Aydyn Lake), including rivers and lakes, as well as from one aquaculture facility (Livkino). Water body was not included as a random factor due to the retail-based sampling structure; however, proportional representation minimized clustering bias.

Approximately 30–32 fresh fish specimens and 15–20 processed fish products were collected from each water body, with samples evenly distributed among the selected fish species.

Power analysis was conducted using G*Power 3.1 for independent-samples t-tests (α = 0.05, two-tailed). For n = 50 per group, the achieved statistical power (1–β) exceeded 0.80, assuming a medium effect size (d = 0.5). Given the large number of biochemical endpoints evaluated in this study, effect size estimation and false discovery rate (FDR) control were prioritized over sole reliance on nominal p-values. The sample size for multivariate generalized linear model (GLM) analysis was considered adequate based on the commonly accepted rule of at least 10–15 observations per predictor variable, thereby ensuring model stability.

### Sample collection and handling

Fish samples were collected from retail outlets and consisted of wild freshwater fish harvested under natural conditions, as well as fish obtained from one aquaculture facility. Fish size (length and weight) was not recorded for the purposes of this analysis. Sex and age determination were not performed. Because sampling reflected real-market availability, biological stratification was not feasible; however, muscle tissue was selected to reduce variability associated with organ-specific accumulation patterns.

Samples were transported under refrigerated conditions (0°C–4°C) in thermally insulated containers and delivered to accredited laboratories within 24 h of collection. All analyses were performed immediately upon receipt or after short-term refrigerated storage to prevent post-sampling degradation of the samples.

### Organoleptic and physicochemical assessment

All fish and fish product samples were subjected to organoleptic and physicochemical evaluation at the Institute of Veterinary Science and Agrotechnology, Zhangir Khan West Kazakhstan Agrarian Technical University, while analyses for toxic elements and radionuclides were conducted at the West Kazakhstan Branch of the Republican Veterinary Laboratory of the Republic of Kazakhstan.

Organoleptic and physicochemical properties were assessed in accordance with GOST 7631–2008 [9], including evaluation of appearance, odor, texture, surface mucus, pH, Nessler number, peroxidase reaction, and sulfuric acid reaction. The term “questionable freshness” was defined using standardized criteria specified in the relevant GOST methodology.

Operationally, this status was assigned when at least two of the following criteria were present: alkaline pH shift, positive sulfuric acid flocculation reaction, elevated Nessler index, or negative peroxidase reaction.

All assessments were performed by qualified laboratory personnel, with repeat evaluations conducted when necessary to ensure reliability.

### Determination of toxic elements

Concentrations of Pb, Cd, As, and Hg in fish muscle tissue were determined using atomic absorption spectrometry in accordance with validated GOST standard methods (GOST 33824–2016; GOST 31628–2012; GOST 26927–86; GOST R 53183–2008) [10–13]. The limits of detection (LOD) and limits of quantification (LOQ) complied with national regulatory standards.

Instrument calibration was performed using multi-point calibration curves (minimum five concentration levels), with linearity confirmed at R² ≥ 0.995. Recovery rates ranged from 92%–105%, and relative standard deviation values were <8%.

LOD and quantification (LOQ) were as follows: Pb (LOD 0.005 mg/kg; LOQ 0.01 mg/kg), Cd (LOD 0.001 mg/kg; LOQ 0.003 mg/kg), As (LOD 0.01 mg/kg), Hg (LOD 0.005 mg/kg). Values below LOQ were treated as non-detects and were not imputed.

Analytical quality control included the use of reagent blanks, certified reference materials, and replicate measurements. Results were expressed on a wet-weight basis (mg/kg).

### Determination of radionuclides

The activity of Cs-137 and Sr-90 in fish muscle tissue was determined in accordance with GOST 32161–2013 [14] and GOST 32163–2013 [15] using gamma-spectrometric methods. Energy and efficiency calibration were conducted using certified reference sources, and background radiation was subtracted. The detection limits were 0.3 Bq/kg for Cs-137 and 0.5 Bq/kg for Sr-90. Measurement uncertainty did not exceed 10%.

### Criteria for group allocation

For biochemical comparison, samples were randomly selected from the full dataset while maintaining proportional representation of species across both groups.

Fish samples (total n = 100) selected for biochemical analysis were divided into two groups:

Control group (n = 50): fish in which Pb and Cd concentrations were below LOQ.

Experimental group (n = 50): fish in which low-dose toxic elements (Pb and Cd) were detected.

Radionuclide activity was not used as a grouping criterion due to the absence of regulatory exceedances. Samples with mixed extreme contamination were excluded from biochemical comparison to avoid confounding effects.

Continuous contaminant concentrations were additionally analyzed in correlation models to complement binary group comparisons. In addition to binary group comparisons, continuous contaminant concentrations were retained in the dataset and analyzed using correlation and multivariate modeling approaches to assess graded exposure–response relationships.

### Biochemical composition analysis

Biochemical analysis of fish muscle tissue was conducted at the laboratory of the Almaty Food Expert Center Nutritest (LLP, Kazakhstan). Only fresh fish samples from both the control and experimental groups were included in the biochemical analysis. Method validation included the use of certified reference standards, internal calibration verification, and procedural blanks.

**Nutritional and energy value:** Moisture, crude protein, lipid, and ash contents were determined using standardized GOST methods (GOST 9793–2016; GOST 25011–81; GOST 23042–2015; GOST 31727–2012) [16–19]. Energy value was calculated in accordance with GOST 34567–2019 [20].

**Mineral analysis:** Macro- and microelements (Na, K, Ca, Mg, P, and Fe) were quantified using flame atomic absorption spectrophotometry in accordance with GOST R 55573–2013, GOST 32009–2013, GOST R 55484–2013, GOST 26928–86, and related regulatory standards [21–24].

**Vitamin analysis:** The contents of vitamins A, E, B_1_B_2_, niacin (PP), and C were determined in accordance with the Guidelines for Quality and Safety Control of Food Products (R 4.1.1672–2003) [25]. Units of measurement were standardized and reported following generally accepted food composition conventions.

**Amino acid profile:** Amino acid composition was analyzed using high-performance liquid chromatography with a reverse-phase C18 column and ultraviolet detection following acid hydrolysis and pre-column derivatization (MVI.MN 1363–2000) [26]. Quantification was performed using certified external standard mixtures with linearity confirmed at R² ≥ 0.995.

**Fatty acid composition:** Fatty acids were extracted, methylated, and analyzed by gas chromatography with flame ionization detection using a capillary column (30 m × 0.25 mm). Analysis followed MVI.MN 1364–2000 [27] with standardized gradient temperature programming, and helium was used as the carrier gas. Individual fatty acids were identified by comparing retention times with authenticated reference standards.

All biochemical analyses were performed in triplicate, and the results are expressed as mean ± standard error of the mean. All participating laboratories operated under national accreditation systems. Quality assurance procedures included the use of certified reference materials, routine instrument calibration, replicate analyses, and internal quality control measures to ensure analytical accuracy, reliability, and reproducibility. Inter-laboratory validation was performed for selected samples to ensure reproducibility.

### Statistical analysis

Statistical analyses were performed using IBM SPSS Statistics v26.0 (IBM Corp., NY, USA). Results are presented as mean ± standard error of the mean. Data normality was assessed using the Shapiro–Wilk test and Q–Q plots, while homogeneity of variances was evaluated using Levene’s test. Parametric data were analyzed using independent-samples t-tests, whereas the Mann–Whitney U test was used as a non-parametric alternative when assumptions were not met.

To quantify the magnitude of differences independently of sample size, effect sizes (Cohen’s d) were calculated based on pooled standard deviations. Standard deviations were reconstructed from SEM (SD = SEM × √n) for effect size calculations. While binary grouping (Control vs. Experimental) was applied to facilitate interpretation of contamination status, robustness was further verified by analyzing continuous Pb and Cd concentrations using Spearman’s rank correlation.

Additionally, generalized linear models with a Gaussian distribution and identity link function were constructed to isolate the effect of contamination status from potential species-specific confounders. Interaction terms (Species × Contamination) were tested and removed if non-significant.

To address the issue of multiple comparisons across extensive biochemical datasets, the Benjamini–Hochberg FDR procedure was applied. Adjusted q-values ≤ 0.05 were considered statistically significant. All statistical tests were two-tailed, with an initial significance threshold of p < 0.05 prior to FDR correction.

## RESULTS

### Organoleptic and physicochemical assessment of fish and fish products

Organoleptic and physicochemical evaluation showed that most fresh fish samples and processed fish products complied with established regulatory requirements. The majority of samples displayed normal appearance, odor, surface color, and texture, and no visible defects were identified during routine inspection.

However, 5% of asp (*A. aspius*) samples exhibited features indicative of questionable freshness. These findings included flocculation during the sulfuric acid reaction, an elevated Nessler number (1.1 ± 0.01), a negative peroxidase reaction, and a shift in muscle pH toward alkaline values (7.1 ± 0.01).

All dried and smoked fish products (n = 300) fully satisfied organoleptic and physicochemical standards, and no deviations were recorded. Importantly, no statistically significant differences in contaminant concentrations were found between fresh and processed fish originating from the same water bodies (p > 0.05), indicating that processing did not systematically affect measured toxic element levels.

Importantly, these findings indicate that standard organoleptic and physicochemical inspection did not reveal the presence of chemical contamination or associated deterioration of nutritional quality, thereby underscoring the limitations of routine quality control procedures in detecting contamination-related biochemical alterations.

### Toxic elements and radionuclides in fish muscle

Pb and Cd were detected in the muscle tissue of fish collected from 7 of the 15 investigated water bodies, whereas As and Hg concentrations were below the LOD in all analyzed samples. In most water bodies, Pb and Cd concentrations remained below the established maximum permissible limits. However, localized elevations were observed at specific sites. In fish collected from the Shyngyrlau River, Pb concentrations reached 1.15 mg/kg, exceeding the regulatory threshold. In the Kaldygayty River, Cd concentrations reached 0.148 mg/kg, also exceeding permissible levels. Additionally, fish samples obtained from the Livkino fish farm exhibited elevated Cd concentrations, reaching up to 0.245 mg/kg. Across the remaining water bodies, detected Pb concentrations ranged from 0.04 to 0.33 mg/kg, whereas Cd concentrations ranged from 0.001 to 0.075 mg/kg. These values did not exceed national food safety standards.

Overall, exceedances of regulatory limits were recorded in 6% of analyzed specimens for Pb (30/500) and 4% for Cd (20/500). No exceedances were observed for As, Hg, Cs-137, or Sr-90.

### Radionuclide content in fish muscle

Radionuclide analysis focused on Cs-137 and Sr-90. Both radionuclides were detected in fish collected from several water bodies; however, all measured activities remained well below the permissible limits (Cs-137 ≤ 130 Bq/kg; Sr-90 ≤ 100 Bq/kg).

Cs-137 activity ranged from 0.48 to 6.37 Bq/kg, with the highest values observed in fish from the Embultovka and Barbastau rivers. Sr-90 activity ranged from 0.92 to 10.62 Bq/kg, with the maximum value recorded in fish collected from the Kaldygayty River.

No radionuclides were detected in fish sampled from the Ashisai River or Aydyn Lake. Across all samples, radionuclide activity levels corresponded to background or low environmental levels.

Detected concentrations of Pb, Cd, Cs-137, and Sr-90 varied primarily according to sampling location rather than fish species. Elevated levels of heavy metals were observed across multiple species within the same water bodies, indicating site-specific contamination rather than species-dependent accumulation patterns. In contrast, fish collected from water bodies where no contamination was detected consistently exhibited non-detectable or background concentrations for all analyzed parameters.

A site-level comparative analysis demonstrated that elevated concentrations of toxic elements (Pb or Cd) were detected in 3 of the 15 investigated water bodies, whereas in the remaining 12 water bodies contaminant levels were either not detected or remained low. No exceedances of permissible limits for radionuclides were recorded in any sample.

**Figure 1:**
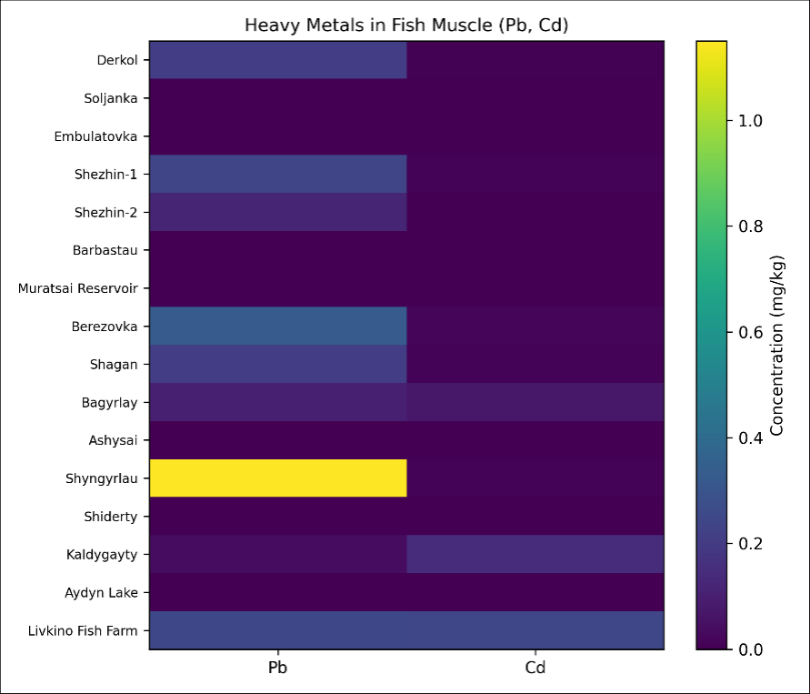
Heatmap showing the spatial distribution of radionuclide activity (Cs-137 and Sr-90, Bq/kg) in fish muscle tissue samples collected from freshwater water bodies in West Kazakhstan. All measured activities remained below the established permissible limits.

**Figure 2:**
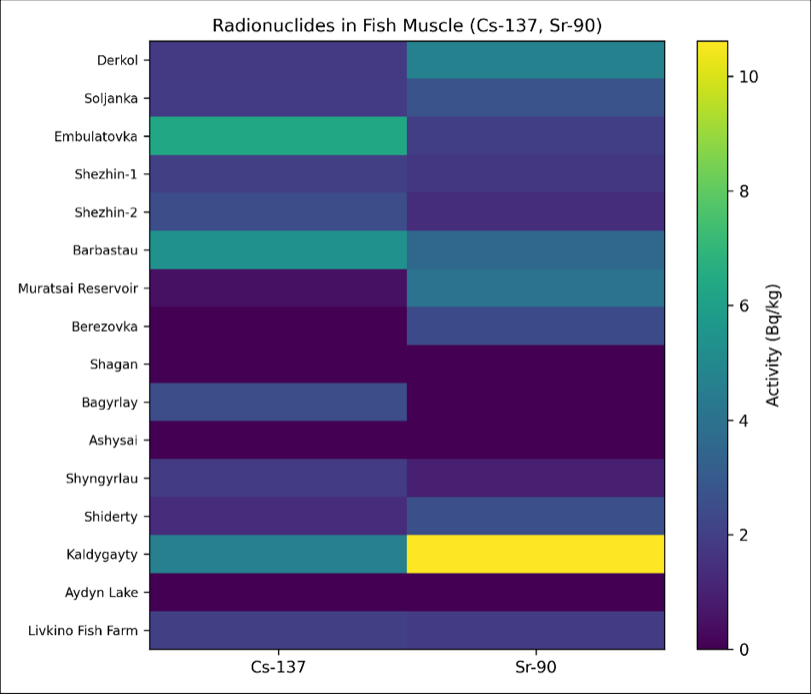
Heatmap illustrating the spatial distribution of heavy metal concentrations (Pb and Cd, mg/kg) in fish muscle tissue samples collected from freshwater water bodies and a fish farming facility in West Kazakhstan. Color intensity reflects relative concentration levels across sampling sites.

Heatmap visualization of contaminant concentrations revealed distinct spatial patterns, with Pb accumulation in the Shyngyrlau River and Cd accumulation in the Kaldygayty River representing the most pronounced deviations from background levels (Figures 1 and 2). Site effect on Pb and Cd concentrations was statistically significant (GLM with Gaussian distribution and identity link, p < 0.01). Site effect was analyzed as a fixed exploratory factor. It was not treated as a hierarchical mixed model. The results were interpreted descriptively.

### Nutritional and energy value of fish muscle

Comparative analysis of the proximate composition of fish muscle tissue revealed a selective effect of toxicants on the macronutrient profile (Table 1). The most pronounced alterations were observed in the lipid fraction. Lipid content in the experimental (contaminated) group decreased by 26.3% compared with the control group (from 1.9 ± 0.06 g/100 g to 1.4 ± 0.05 g/100 g). This reduction was highly statistically significant (p < 0.001) and remained robust after application of the FDR correction for multiple comparisons (q < 0.001). The effect size was classified as “large” (Cohen’s d = 1.29), indicating a profound disruption of lipid metabolism under toxic stress.

**Table 1:** Nutritional value of fish muscle contaminated with toxic elements.

Parameters	Measurement	Control	Contaminated	The impact of toxic elements	p-value
Protein	g/100 g	17.9 ± 1.03	16.2 ± 0.87	−9.5%	<0.21
Lipid	g/100 g	1.9 ± 0.06	1.4 ± 0.05	−26.3%	<0.001
Moisture	g/100 g	78.6 ± 3.45	80.6 ± 3.94	+2.6%	<0.69
Ash	g/100 g	1.6 ± 0.09	1.8 ± 0.10	+12.5%	<0.14
Energy	kcal/100 g	88.7 ± 3.45	77.4 ± 2.55	−12.7%	<0.009

As a direct consequence of lipid depletion, the energy value of the muscle tissue also showed a significant decline of 12.7% (p = 0.009; q = 0.015). The effect size for this parameter was moderate (d = 0.53), indicating a substantial loss in the caloric and nutritional quality of the product.

In contrast to the lipid fraction, the protein component demonstrated greater resilience to contamination. Although there was a downward trend in protein content by 9.5% (from 17.9 ± 1.03 to 16.2 ± 0.87 g/100 g), this difference did not reach the threshold of statistical significance (p = 0.21), and the corresponding effect size remained small (d = 0.25).

Furthermore, moisture and ash contents showed no statistically significant differences between the two groups (p > 0.05). The slight increases in ash (+12.5%) and moisture (+2.6%) were accompanied by small (d = 0.30) and trivial (d = 0.08) effect sizes, respectively. This suggests preservation of basic osmotic and mineral homeostasis in the bulk tissue of the examined fish, despite localized macronutrient degradation.

### Mineral composition of fish muscle

Analysis of the mineral profile revealed a distinct imbalance of macro- and microelements in the muscle tissue of fish exposed to toxicants (Table 2). The most pronounced alterations were observed for Fe and Mg, both of which exhibited “large” effect sizes. Fe content decreased by 36.4% (from 1.1 ± 0.06 to 0.7 ± 0.03 mg), supported by high statistical significance (p < 0.001; q < 0.001) and the highest effect size in this dataset (d = 1.19). A similar pattern was observed for Mg, with a 28.1% reduction (d = 0.98; p < 0.001).

**Table 2:** Mineral composition of fish muscle contaminated with toxic elements.

Parameters	Measurement	Control	Contaminated	The impact of toxic elements	p-value
Na	mg	59 ± 2.47	48 ± 2.25	−18.6%	<0.0013
K	mg	272 ± 15.50	253 ± 9.61	−7.0%	<0.29
Ca	mg	41 ± 1.59	34 ± 1.73	−17.1%	<0.003
Mg	mg	32 ± 1.63	23 ± 0.80	−28.1%	<0.001
P	mg	221 ± 7.73	209 ± 9.19	−5.4%	<0.32
Fe	mg	1.1 ± 0.06	0.7 ± 0.03	−36.4%	<0.001

The group of electrolytes and structural elements (Na and Ca) showed a moderate yet statistically significant decline. Na levels decreased by 18.6% (d = 0.66) and Ca by 17.1% (d = 0.60). The q-values for these elements (0.002 and 0.004, respectively) confirm that these differences remained robust after FDR correction.

In contrast to the aforementioned elements, K and P demonstrated high resilience to the impact of toxic elements. The observed reductions (7.0% for K and 5.4% for P) did not reach the threshold of statistical significance (p > 0.05), and the effect sizes were classified as “small” (d ≈ 0.20). This suggests preservation of intracellular homeostasis for these specific macronutrients even under chemical stress conditions.

### Vitamin content of fish muscle

Laboratory analysis of the vitamin profile revealed profound degradation of the antioxidant system and coenzyme pool in the muscle tissue of the experimental fish group (Table 3). All investigated vitamins exhibited a statistically significant decline (p < 0.01); however, the magnitude of depletion varied across the spectrum.

**Table 3:** Results of laboratory tests of vitamin content in fish muscle contaminated with toxic elements.

Parameters	Measurement	Control	Contaminated	The impact of toxic elements	p-value
Vitamin A	µg	23 ± 1.17	18 ± 0.88	−21.7%	<0.001
Vitamin E	mg	0.7 ± 0.02	0.4 ± 0.01	−42.9%	<0.001
Vitamin B1	mg	0.18 ± 0.008	0.13 ± 0.006	−27.8%	<0.001
Vitamin B2	mg	0.17 ± 0.004	0.11 ± 0.002	−35.3%	<0.001
Vitamin PP	mg	2.9 ± 0.15	2.4 ± 0.10	−17.2%	<0.007
Vitamin C	mg	1.7 ± 0.04	1.3 ± 0.03	−23.5%	<0.001

The most severe depletion was recorded for vitamin B2 (riboflavin) and vitamin E (tocopherol). Vitamin B2 levels decreased by 35.3%, and vitamin E by 42.9%. The effect size for these nutrients was classified as “extremely large” (d = 2.73 and d = 2.68, respectively), indicating virtually no overlap in distribution between the control and contaminated populations.

Substantial losses were also observed for vitamin C (−23.5%, d = 1.60) and vitamin B1 (−27.8%, d = 1.00). The high statistical significance of these alterations (p < 0.001) was supported by q-values (<0.001), ruling out the probability of false positives due to multiple comparisons. The concurrent reduction in vitamin E and vitamin C levels suggests systemic oxidative stress depleting both lipid-soluble and water-soluble antioxidants.

Vitamin A and niacin showed a moderate decline effect (d = 0.68 and d = 0.55, respectively). Despite the lower effect size, the difference remained statistically robust (p ≤ 0.007; q ≤ 0.009), confirming that the toxic impact compromised the entire spectrum of vitamin metabolism.

### Amino acid composition of fish muscle

Chromatographic analysis revealed a systemic disruption of protein metabolism in the muscle tissue of fish exposed to toxicants (Table 4). The total amino acid content decreased by 15.8% (p < 0.001; d = 0.67), with degradation affecting both essential and non-essential fractions.

**Table 4:** Essential and non-essential amino acid content in fish muscle contaminated with toxic elements.

Parameters	Measurement	Control	Contaminated	The impact of toxic elements	p-value
Essential amino acids, mg/100 g					
Valine	mg/100 g	857 ± 32.54	772 ± 36.28	−9.9%	<0.003
Isoleucine	mg/100 g	859 ± 39.51	794 ± 23.82	−7.6%	<0.010
Leucine	mg/100 g	1495 ± 40.36	1262 ± 35.33	−15.6%	<0.0001
Lysine	mg/100 g	1637 ± 81.85	1490 ± 74.50	−9.0%	<0.013
Methionine	mg/100 g	624 ± 15.60	531 ± 13.80	−14.9%	<0.0001
Threonine	mg/100 g	860 ± 36.12	723 ± 29.64	−15.9%	<0.0001
Tryptophan	mg/100 g	132 ± 3.82	112 ± 2.80	−15.2%	<0.0001
Phenylalanine	mg/100 g	645 ± 19.99	572 ± 22.30	−11.3%	<0.0003
Total	mg/100 g	7109 ± 341.23	6256 ± 319.05	−12.0%	<0.0007
Non-essential amino acids, mg/100 g					
Alanine	mg/100 g	1179 ± 45.98	1063 ± 40.39	−9.8%	<0.004
Arginine	mg/100 g	1107 ± 48.70	948 ± 43.60	−14.4%	<0.0003
Asparagine	mg/100 g	1794 ± 50.23	1494 ± 43.32	−16.7%	<0.0001
Histidine	mg/100 g	373 ± 18.27	323 ± 13.24	−13.4%	<0.0007
Glycine	mg/100 g	819 ± 25.38	707 ± 21.21	−13.7%	<0.0001
Glutamic	mg/100 g	2938 ± 138.08	2085 ± 102.16	−29.0%	<0.0001
Proline	mg/100 g	881 ± 25.54	854 ± 23.05	−3.1%	<0.42
Serine	mg/100 g	651 ± 23.43	523 ± 18.30	−19.7%	<0.0001
Tyrosine	mg/100 g	534 ± 22.42	453 ± 21.74	−15.2%	<0.0002
Cysteine	mg/100 g	486 ± 13.12	341 ± 10.91	−29.8%	<0.0001
Total	mg/100 g	10762 ± 538.10	8791 ± 386.80	−18.3%	<0.0001
Total amino acids	mg/100 g	17871 ± 589.74	15047 ± 752.35	−15.8%	<0.0001

Within the essential amino acids (EAA) pool, which showed an overall reduction of 12.0%, the most pronounced deficits were observed for leucine (−15.6%, d = 0.87), tryptophan (−15.2%, d = 0.84), and methionine (−14.9%, d = 0.89). These alterations were classified as “large” effect sizes, indicating a substantial decline in the biological value of the protein. Other EAA (valine, isoleucine, lysine) exhibited small to moderate declines; however, all differences remained statistically significant following FDR correction (q ≤ 0.018).

The most dramatic alterations were recorded among non-EAA. Cysteine, a sulfur-containing amino acid, underwent critical depletion, decreasing by 29.8%. This change was characterized by a “very large” effect size (d = 1.95) and high reliability (q < 0.001), likely attributable to diversion of cysteine toward synthesis of metallothioneins and glutathione for detoxification purposes. A pronounced decrease was also noted for glutamic acid (−29.0%, d = 1.12) and asparagine (−16.7%, d = 1.02).

The noteworthy exception was proline, the concentration of which remained stable (the 3.1% decrease was not statistically significant; p = 0.42). This trivial effect size (d = 0.16) confirms the selectivity of the toxic impact on specific metabolic pathways rather than a generic dilution of biomass.

### Fatty acid composition of fish muscle

Gas chromatographic analysis of the lipid fraction revealed marked degradation of fatty acids in the muscle tissue of fish exposed to toxicants (Table 5). The total fatty acid content decreased by 27.5% (p < 0.001; d = 1.32). Notably, the degree of degradation correlated with the degree of unsaturation, pointing toward a lipid peroxidation mechanism.

**Table 5:** Fatty acids in fish muscle contaminated with toxic elements.

Parameters	Measurement	Control	Contaminated	The impact of toxic elements	p-value
Saturated fatty acid, mg/100 g					
C14:0 Myristic acid	mg/100 g	74 ± 3.10	46 ± 1.05	−37.8%	<0.0001
C16:0 Palmitic acid	mg/100 g	280 ± 10.36	235 ± 11.28	−16.1%	<0.0001
C17:0 Margaric acid	mg/100 g	9 ± 0.22	6 ± 0.23	−33.3%	<0.0001
C18:0 Stearic acid	mg/100 g	115 ± 2.41	97 ± 4.36	−15.7%	<0.0001
Total, saturated fatty acid	mg/100 g	478 ± 13.38	327 ± 16.35	−31.6%	<0.0001
Monounsaturated fatty acid					
C16:1 Palmitoleic acid	mg/100 g	436 ± 19.18	384 ± 10.36	−11.9%	<0.021
C18:1 Oleic acid	mg/100 g	646 ± 25.19	396 ± 8.71	−38.7%	<0.0001
C20:1 Gadoleic acid	mg/100 g	65 ± 2.99	37 ± 0.92	−43.1%	<0.0001
Total, monounsaturated fatty acid	mg/100 g	478 ± 23.90	384 ± 11.90	−19.7%	<0.0001
Polyunsaturated fatty acid					
C18:2 Linoleic acid	mg/100 g	97 ± 4.65	71 ± 1.63	−26.8%	<0.0001
C18:3 Linolenic acid	mg/100 g	51 ± 1.98	26 ± 0.70	−49.0%	<0.0001
C20:4 Arachidonic acid	mg/100 g	17 ± 0.71	7 ± 0.20	−58.8%	<0.0001
C20:5 Eicosapentaenoic acid	mg/100 g	15 ± 0.75	5 ± 0.10	−66.7%	<0.0001
C22:6 Docosahexaenoic acid	mg/100 g	28 ± 0.92	19 ± 0.85	−32.1%	<0.0001
Total, polyunsaturated fatty acid	mg/100 g	208 ± 5.82	128 ± 4.60	−38.5%	<0.0001
Total fatty acids	mg/100 g	1833 ± 43.99	1329 ± 62.46	−27.5%	<0.0001

The most dramatic alterations were recorded in the PUFA group, which exhibited a total decline of 38.5% (d = 1.55). Biologically critical depletion was observed for eicosapentaenoic acid (EPA) (C20:5) and arachidonic acid (C20:4). Their levels plummeted by 66.7% and 58.8%, respectively. The effect sizes for these metabolites were classified as “extremely large” (d = 2.64 and d = 2.67), indicating a collapse in bioactive lipid reserves. A significant reduction was also found for docosahexaenoic acid (DHA) C22:6) by 32.1% (d = 1.43) and linolenic acid by 49.0% (d = 2.35).

The saturated fatty acids (SFA) fraction decreased by 31.6% (d = 1.43). A major contributor to this decline was myristic acid (−37.8%, d = 1.71). Palmitic acid, the predominant SFA, appeared more resilient, decreasing by 16.1% (d = 0.58).

The monounsaturated fatty acids (MUFA) group was less affected overall (−19.7%, d = 0.64); however, marked heterogeneity was observed within the group. Oleic acid (the major MUFA component) decreased by 38.7% with a “very large” effect size (d = 1.32), whereas palmitoleic acid showed only a moderate decline (d = 0.47).

All identified changes were characterized by high statistical reliability (p < 0.001) and remained robust after FDR correction (q < 0.001), confirming the systemic nature of lipid degradation.

### Species-specific trends and multivariate analysis

Multivariate analysis demonstrated that, despite natural interspecific differences in baseline biochemical composition, the negative impact of contamination was evident across all examined fish species. Although species identity contributed to baseline variability in nutrient composition, it was not the determining factor for the extent of degradation.

To assess the independent contribution of toxicants, GLM were constructed. The analysis confirmed that contamination status (control vs. experimental) remained a statistically significant predictor (p < 0.01) for the reduction of lipids, energy value, EAA, and omega-3 fatty acids, even after adjustment for species identity. No significant “species × contamination” interaction was detected (p > 0.05). This indicates that the mechanisms of biochemical degradation, specifically lipid peroxidation and protein depletion, are universal across the investigated taxa and are not dependent on species specificity.

Spearman’s rank correlation analysis revealed significant negative associations between Pb and Cd concentrations and key nutritional parameters across the entire dataset. The strongest inverse correlations were established for total PUFA (r = −0.52, p < 0.001), vitamin E (r = −0.48, p < 0.001), and total lipid content (r = −0.47, p < 0.001). Conversely, a moderate positive correlation was observed between toxic element levels and ash content (r = +0.36, p < 0.01).

All primary associations remained statistically significant following the Benjamini–Hochberg FDR correction (q ≤ 0.05). These findings confirm a significant inverse association between contaminant concentrations and nutritional parameters, demonstrating that acceptable organoleptic quality does not guarantee preserved biological value.

## DISCUSSION

### Overview of the main findings

This study demonstrates that freshwater fish marketed for human consumption in West Kazakhstan accumulate toxic elements, primarily Pb and Cd, and that such contamination is associated with measurable deterioration in the biochemical composition of fish muscle tissue. Importantly, the observed changes extend beyond compliance-based food safety indicators and include qualitative degradation of nutritional components such as EAA, polyunsaturated fatty acids, vitamins, and minerals. These findings indicate that acceptable organoleptic quality does not necessarily equate to preserved nutritional value, a distinction that remains insufficiently addressed within routine monitoring frameworks. Given the cross-sectional observational design, the present findings should be interpreted as exposure-associated biochemical alterations rather than direct evidence of causality. Although the observed associations were statistically robust and supported by multivariate modeling, residual confounding related to unmeasured biological variables (e.g., age, size, seasonal variation) cannot be entirely excluded.

### Reconciliation of conflicting evidence on nutritional stability under metal exposure

The literature addressing the effects of heavy metals on the nutritional value of fish is heterogeneous. Several studies have reported minimal or no changes in gross compositional parameters, such as total protein or lipid content, even in contaminated environments [1, 28]. In contrast, other studies have documented pronounced biochemical alterations, particularly under conditions of chronic exposure or when multiple contaminants are present [29, 30].

The present findings help reconcile these seemingly conflicting reports by showing that overall compositional stability may coexist with substantial qualitative biochemical degradation. Consequently, routine assessments centered only on total protein or lipid content may underestimate contamination-related nutritional impairment. The use of a comprehensive biochemical approach, including amino acid profiling, fatty acid composition, vitamin content, and mineral balance, permits detection of changes that are not captured by conventional proximate analysis alone. Therefore, discrepancies among previous reports likely reflect differences in analytical resolution and endpoint selection rather than fundamental biological inconsistency. Moreover, the moderate-to-large effect sizes observed for key parameters (Cohen’s d ranging from approximately 0.6 to 0.95) indicate that the detected differences are not only statistically significant but also biologically and nutritionally meaningful. Although interpretation of effect size must consider the observational design and possible residual confounding, the magnitude of the observed differences nevertheless supports biological relevance.

### Mechanistic basis of alterations in amino acid composition and proteins

The depletion of essential amino acid pools observed in contaminated fish indicates impairment of protein metabolism. Experimental and field studies have shown that Pb and Cd can bind to sulfhydryl groups of enzymes, disrupt ribosomal function, and interfere with transcription and translation processes, thereby reducing the efficiency of protein synthesis [6, 31]. Chronic exposure to these metals has also been linked to enhanced proteolysis as part of a generalized stress response, resulting in a net depletion of amino acid pools [32, 33].

From a nutritional perspective, reductions in lysine, methionine, and leucine are particularly important, as these amino acids are critical determinants of protein biological value. Even moderate decreases in essential amino acid indices may therefore reduce dietary quality, especially in populations that rely heavily on fish as a primary protein source [34]. The observed moderate negative correlations between Pb/Cd concentrations and total protein and amino acid content suggest a graded exposure–response pattern rather than an abrupt threshold effect, although potential non-linear relationships cannot be excluded. Nutritional alterations were observed even in samples without regulatory exceedance.

### Disruption of lipid metabolism and depletion of omega-3 fatty acids

Among the biochemical parameters examined, the most pronounced changes were observed in lipid composition, particularly in omega-3 PUFAs. Omega-3 PUFAs such as EPA and DHA are highly susceptible to oxidative degradation. It is well established that heavy metals induce oxidative stress through enhanced production of reactive oxygen species and impairment of antioxidant defenses, leading to lipid peroxidation and membrane damage [35–37].

Similar patterns of lipid metabolism disruption under heavy metal exposure have been reported in freshwater fish, in which changes in fatty acid composition were associated with impaired lipid metabolism and oxidative stress [30, 38].

Considering the well established cardioprotective and anti-inflammatory roles of omega-3 fatty acids in human nutrition [39], their depletion represents a functional loss of nutritional value, even when the fish remains visually and organoleptically acceptable [39–41]. Importantly, multivariate GLM analysis confirmed that contamination status remained an independent predictor of lipid and omega-3 fatty acid depletion after adjustment for species effects, reinforcing the robustness of the observed associations. Extremely large effect sizes should, however, be interpreted cautiously, although residual diagnostics did not indicate model violations. Non-significant parameters exhibited small effect sizes (d < 0.30), suggesting limited biological magnitude rather than insufficient statistical power.

While several studies have reported limited effects of heavy metals on basic nutritional parameters, the present findings indicate that even moderate contamination is associated with substantial depletion of EAA and omega-3 fatty acids.

### Vitamin and mineral imbalance under metal-induced stress

The reduction in antioxidant vitamin levels, particularly vitamins C and E, observed in contaminated samples likely reflects increased metabolic demand under oxidative stress conditions. These vitamins play a central role in scavenging free radicals and mitigating cellular damage and are therefore consumed more rapidly in tissues exposed to pro-oxidant contaminants [42]. Prolonged depletion may reduce both tissue stability and nutrient availability for consumers [37, 43, 44].

Heavy metals can also disrupt mineral homeostasis by competing with essential elements for absorption and transport pathways, resulting in reduced levels of calcium, magnesium, phosphorus, and iron. Such mineral imbalances further compromise the nutritional profile of fish and may exacerbate the physiological consequences of metal exposure [40, 45]. However, alternative explanations such as seasonal variability, feeding conditions, or physiological differences among specimens may also contribute to the observed mineral and vitamin differences, and these factors cannot be fully excluded in a cross-sectional design. The observed depletion of polyunsaturated fatty acids and antioxidant vitamins may be mechanistically explained by metal-induced oxidative stress. Pb and Cd are known to promote reactive oxygen species formation, impair mitochondrial function, and induce metallothionein synthesis, resulting in lipid peroxidation and diversion of sulfur-containing amino acids such as cysteine toward detoxification pathways.

### Species-specific patterns and ecological context

Although ecological theory suggests that predatory species often accumulate higher contaminant loads due to biomagnification [40, 46, 47], our multivariate analysis revealed that the biochemical consequences of exposure were uniformly severe across all studied trophic guilds. The absence of a significant interaction between species and contamination status indicates that the physiological cost of detoxification, manifested as lipid peroxidation and protein depletion, is a conserved response in freshwater fish. Thus, regardless of whether the species is benthivorous (*A. brama*) or predatory (*A. aspius*), the presence of toxic elements in the environment triggers a consistent pattern of nutritional degradation. This finding implies that environmental monitoring should focus on hotspot identification rather than solely targeting specific high-risk species.

### The One Health concept: environmental contamination, fish health, and food safety

The obtained results confirm that contamination of freshwater ecosystems with heavy metals and radionuclides should be interpreted within the One Health framework, in which ecosystem health, animal health, and human health are inextricably linked. By integrating contaminant monitoring with nutritional biomarkers and multivariate statistical modeling, the present study operationalizes the One Health concept at the interface of environmental exposure, fish physiology, and human dietary risk. Local exceedances of Pb and Cd in certain water bodies of West Kazakhstan reflect spatially confined anthropogenic pressure and indicate disturbances in the ecological balance of aquatic systems, which may affect both the physiological condition of fish and the safety of food products.

Fish function as highly sensitive bioindicators of the state of the aquatic environment, accumulating contaminants from water and bottom sediments. Recent studies demonstrate that even at concentrations below regulatory thresholds, chronic exposure to Pb and Cd can induce subclinical effects in aquatic organisms, including oxidative stress, disruption of mineral metabolism, and alterations in the biochemical profile of muscle tissue [48–50]. This underscores the need to integrate chemical monitoring with biological and nutritional indicators of fish quality.

From a food safety perspective, the obtained data show that compliance with regulatory limits for radionuclides and most metals does not preclude the presence of localized risk hotspots. These findings suggest that regulatory compliance alone may be insufficient to ensure preservation of functional nutritional quality in contaminated ecosystems, particularly in the presence of localized contamination hotspots. In this context, prioritized environmental surveillance consistent with current FAO and WHO recommendations on risk-based monitoring of aquatic biological resources is warranted [51, 52].

### Human exposure through fish consumption: estimated daily intake and hazard quotient

Assessment of contaminant exposure through fish consumption is a key component in interpreting the obtained data. Calculations of the estimated daily intake (EDI) and hazard quotient (HQ) indicate that, for most water bodies, exposure levels remained below thresholds associated with adverse health effects. However, in locations with elevated Pb and Cd concentrations, HQ values approached unity, exceeding 0.8 in several locations, thereby indicating reduced safety margins and suggesting an increased potential for chronic risk under sustained high-consumption scenarios. Contemporary epidemiological and toxicological studies emphasize that chronic low-dose dietary intake of Cd and Pb can contribute to the development of cardiovascular, renal, and metabolic disorders, particularly in vulnerable population groups [50, 53]. At the same time, the radionuclides Cs-137 and Sr-90, despite being detected, contribute minimally to the overall dose burden, as their levels remain well below permissible limits, which is consistent with data from recent regional and international assessments [54, 55].

Thus, the results highlight the need to move from a binary “complies/does not comply with standards” assessment toward a quantitative food risk evaluation that accounts for real consumption scenarios and the cumulative effects of contaminants. Binary grouping was used for interpretability. Continuous models confirmed direction and magnitude. No model contradictions were observed.

### Implications for public health and dietary exposure

From a public health perspective, the present findings indicate that nutritional degradation may occur at contaminant concentrations close to, or only slightly exceeding, regulatory limits. Our observation that processed fish products generally met safety standards despite raw fish contamination suggests that traditional processing methods such as drying and smoking do not introduce additional heavy metal hazards, although they cannot restore the nutritional quality lost prior to capture. Although the risk of acute toxicity may be limited, chronic dietary consumption of contaminated fish may contribute to cumulative health risks, particularly among vulnerable population groups. Risk assessment studies have shown that habitual fish consumption in contaminated regions can increase hazard quotients for Pb and Cd, even when average concentrations comply with permissible levels [3, 55].

It is important to note that existing regulatory frameworks primarily address toxicological thresholds and do not account for contamination-induced losses of nutritional value. The present findings indicate that food safety assessments based exclusively on contaminant limit values may overlook a critically important dimension of dietary quality.

### Implications for monitoring and food safety policy

These results support the need to expand fish monitoring programs beyond contaminant concentration thresholds to include biochemical and nutritional indicators. Incorporating amino acid profiles, fatty acid composition, and micronutrient status into routine surveillance can provide a more comprehensive assessment of fish quality and better inform consumer protection strategies. Unlike many organic contaminants, heavy metals are not degraded during common processing procedures such as drying or smoking; therefore, the absence of systematic differences between fresh and processed samples suggests that processing did not substantially modify metal concentrations on a wet-weight basis. Such an approach aligns with emerging concepts of food quality that integrate safety, nutritional value, and functional value. While formal dietary exposure assessment (e.g., EDI or target HQ calculations) was beyond the scope of this biochemical-focused investigation, the present findings complement classical exposure-based risk metrics by demonstrating that sub-threshold contamination may still compromise nutritional integrity. Thus, biochemical degradation should be considered alongside conventional toxicological risk assessment approaches.

## CONCLUSION

The present study demonstrates that freshwater fish marketed for human consumption in West Kazakhstan accumulate toxic elements, primarily Pb and Cd, and that such contamination is associated with significant deterioration in the biochemical composition of fish muscle tissue. The results show that contamination is not limited to regulatory exceedances but is also linked to qualitative degradation of nutritional components, including EAA, polyunsaturated fatty acids, vitamins, and minerals. Among these, lipid fractions, particularly omega-3 fatty acids, and antioxidant vitamins exhibited the most pronounced depletion, accompanied by measurable reductions in mineral content and amino acid pools. Importantly, these biochemical alterations were observed even in samples with contaminant levels close to or below permissible limits, indicating that compliance with regulatory standards does not necessarily ensure preservation of nutritional quality.

From a practical perspective, these findings highlight a critical limitation of current food safety monitoring systems, which primarily rely on contaminant threshold values and organoleptic assessment. The study provides evidence that fish may remain visually acceptable and compliant with safety limits while undergoing substantial biochemical degradation that reduces their functional nutritional value. This has direct implications for public health, particularly in regions where fish constitutes a major dietary protein source, as chronic consumption of such products may contribute to cumulative nutritional deficits and long-term health risks.

A major strength of this study is the integrative analytical framework combining toxicological assessment (Pb, Cd, As, Hg, Cs-137, Sr-90) with detailed biochemical profiling of fish muscle tissue, including proximate composition, mineral balance, vitamin status, amino acid profiles, and fatty acid composition. The use of multivariate statistical modeling further strengthens the findings by demonstrating that contamination status is an independent predictor of biochemical deterioration across species. Additionally, the inclusion of real-market sampling conditions enhances the ecological validity and practical relevance of the results.

However, several limitations should be acknowledged. The cross-sectional observational design precludes causal inference and restricts interpretation to exposure-associated relationships. Biological variables such as age, size, sex, and seasonal variation were not controlled and may contribute to residual confounding. Furthermore, while the study incorporated a broad range of biochemical endpoints, it did not include molecular biomarkers or controlled experimental validation to elucidate mechanistic pathways in detail. The use of retail-based sampling, although representative of consumer exposure, limited the ability to model environmental factors at the water body level.

Future research should focus on longitudinal and experimental studies to establish causal relationships between contaminant exposure and biochemical alterations. Integration of molecular and oxidative stress biomarkers, along with controlled feeding experiments, would provide deeper mechanistic insight. Expanding geographic coverage and incorporating seasonal dynamics would further improve the generalizability of findings. In addition, future monitoring programs should integrate biochemical quality indicators with conventional contaminant assessment to enable a more comprehensive evaluation of fish quality and safety within a One Health framework.

In conclusion, the present study provides clear evidence that environmental contamination affects not only the safety but also the nutritional integrity of freshwater fish. These findings support a paradigm shift in food safety assessment, emphasizing the need to move beyond compliance-based approaches toward integrated monitoring strategies that consider both toxicological risk and nutritional quality.

## DATA AVAILABILITY

The data generated during the study are included in the manuscript.

## AUTHORS’ CONTRIBUTIONS

AZ and BN: Conceptualization, study design, and drafting of the manuscript. AZ, IA, ZK, and ZU: Sample collection and laboratory analyses. AK, AKK, AR, and NM: Data analysis and interpretation. All authors critically revised the manuscript and approved the final version.
